# Selective aneurysmal sac neck-targeted embolization during endovascular repair of abdominal aortic aneurysm with hostile neck anatomy

**DOI:** 10.1186/s13019-024-02550-z

**Published:** 2024-02-04

**Authors:** Lifeng Zhang, Yongjiang Tang, Jiantao Wang, Xianjun Liu, Yang Liu, Wei Zeng, Chunshui He

**Affiliations:** 1 Department of Vascular Surgery, Hospital of Chengdu University of Traditional Chinese Medicine, Chengdu, 610072 Sichuan China; 2 Department of Vascular Disease, Panzhihua Municipal Central Hospital, Panzhihua, Sichuan China; 3 Department of Interventional Radiology and Vascular Surgery, Xichang Municipal Pepole’s Hospital, Xichang, Sichuan China; 4 Department of Interventional Radiology, Leshan Hospital of Traditional Chinese Medicine, Leshan, Sichuan China

**Keywords:** Endovascular aneurysm repair, Hostility, Neck, Anatomy, Endoleak, Therapeutic embolization

## Abstract

**Purpose:**

To evaluate the efficacy and safety of selective aneurysmal sac neck-targeted embolization in endovascular aneurysm repair (EVAR) in patients with a hostile neck anatomy (HNA).

**Materials and methods:**

Between October 2020 and June 2022, patients with an abdominal aortic aneurysm (AAA) and HNA who underwent EVAR with a low-profile stent graft and a selective aneurysmal sac neck-targeted embolization technique were analysed. An HNA was defined by the presence of any of the following parameters: infrarenal neck angulation > 60°; neck length < 15 mm; conical neck; circumferential calcification ≥ 50%; or thrombus ≥ 50%. Before occluding the entire aneurysm during the procedure, a buddy wire was loaded prophylactically into the sac through the contralateral limb side. If a type Ia endoleak (ELIa) occurred and persisted despite adjunctive treatment such as balloon moulding or cuff extension, this preloaded wire could be utilized to enable a catheter to reach the space between the stent graft and sac neck to perform coil embolization. In the absence of ELIa, the wire was simply retracted. The primary outcome of this study was freedom from sac expansion and endoleak-related reintervention during the follow-up period; secondary outcomes included technical success and intraoperative and in-hospital postoperative complications.

**Results:**

Among the 28 patients with a hostile neck morphology, 11 (39.5%) who presented with ELIa underwent intraprocedural treatment involving sac neck-targeted detachable coil embolization. Seventeen individuals (60.7%) of the total patient population did not undergo coiling. All patients in the coiling group underwent balloon moulding, and 2 patients additionally underwent cuff extension. In the noncoiling group, 14 individuals underwent balloon moulding as a treatment for ELIa, while 3 patients did not exhibit ELIa during the procedure. The coiling group showed longer operating durations (81.27 ± 11.61 vs. 70.71 ± 7.17 min, *P* < 0.01) and greater contrast utilization than the noncoiling group (177.45 ± 52.41 vs. 108.24 ± 17.49 ml, *P* < 0.01). In the entire cohort, the technical success rate was 100%, and there were no procedure-related complications. At a mean follow-up of 18.6 ± 5.2 months (range 12–31), there were no cases of sac expansion (19 cases of sac regression, 67.86%; 9 cases of stability, 32.14%) or endoleak-related reintervention.

**Conclusions:**

Selective aneurysmal sac neck-targeted embolization for the treatment of ELIa in AAA patients with an HNA undergoing EVAR is safe and may prevent type Ia endoleak and related sac expansion after EVAR.

## Introduction

Endovascular aneurysm repair (EVAR) has become an acknowledged alternative to open repair (OR) for the treatment of abdominal aortic aneurysms (AAAs) [1, 2]. The shape of the aortic neck is the most important morphological characteristic of infrarenal AAAs for determining the success of endograft repair [3, 4]. To achieve optimal long-term outcomes, aortic endograft manufacturers have defined precise anatomic parameters of the proximal aneurysm neck, such as the length, diameter, and infrarenal angulation, included in the instructions for use (IFU) of endografts. Any aortic neck anatomy that exceeds the parameters specified in the IFU is referred to as a hostile neck anatomy (HNA) [5, 6]. However, in actual clinical practice, up to 40% of EVAR cases involve the presence of an HNA, with acceptable short- and mid-term outcomes [7, 8].

Compared to patients with a favourable neck anatomy, ELIa is substantially more common during the standard EVAR procedure among patients with an HNA [9]. ELIa is known to be related to increased sac pressure and aneurysm expansion and is a significant risk factor for late aneurysm rupture and open conversion. Consequently, intraoperative ELIa management is recommended and usually involves balloon moulding, deployment of covered extension cuffs or large-calibre balloon-expandable stents, application of endoanchors, and endoleak embolization [3, 10].

Recent reports have indicated that the implementation of intraprocedural AAA sac embolization during EVAR has reduced the incidence of type II endoleak [11, 12]. However, there are insufficient data regarding the efficacy of intraprocedural sac neck embolization in eliminating ELIa. We utilized a novel and straightforward technique to selectively embolize the aneurysmal sac neck to halt ELIa in AAA patients with an HNA during EVAR. The purpose of the study was to evaluate the efficacy and safety of this technique.

## Materials and methods

This retrospective cohort study was conducted in accordance with the requirements of the local ethics committee, and all patients provided written informed consent for data collection and analysis at the time of admission.

This study included twenty-eight patients (22 men, 78.57%; overall mean age 71.46 ± 8.98 years) with an AAA and HNA who underwent EVAR using a low-profile endograft at a university hospital and three other municipal hospitals between October 2020 and June 2022. All patients either had contraindications to open surgery or expressed a strong preference for endovascular treatment.

A low-profile stent graft, the Minos™ aortic stent graft (MicroPort® Endovastec™, Shanghai, China), was utilized; the outer diameter was 14 F for the main body and 12 F for the iliac limb prostheses [13]. A patient was identified as having an HNA if the aortic neck morphology fell outside the IFU of the endograft or met any of the following parameters, in accordance with expert opinion [2, 4, 5]: infrarenal neck angulation > 60°; neck length < 15 mm; conical neck; circumferential calcification ≥ 50%; or thrombus ≥ 50%. Software (EndoSize, Therenva SAS, Paris, France) was utilized to conduct anatomic measurements and image analysis.

The inclusion criteria for this study were as follows: maximal aneurysm diameter ≥ 5.5 cm; and at least one aspect of the anatomy of the aneurysmal neck meeting the specified criteria for an HNA in this study. The exclusion criteria encompassed ruptures requiring emergency repair, suspected infection-related aneurysms, and other aortic pathologies, such as saccular aneurysms, penetrating aortic ulcers, or dissections.

### Procedural details

All procedures were conducted in the interventional radiology suite by two experienced physicians who had performed over one hundred conventional EVAR procedures. The aortic bifurcated endograft was ≥ 15% oversized relative to the aneurysmal neck, as measured by preoperative computed tomography angiography (CTA). Under local or general anaesthesia, both femoral access routes were established utilizing the “preclose technique” [14] with one Perclose ProGlide (Abbott Laboratories, Illinois, USA) suture-mediated closure device. After the main body was released and the contralateral gate was successfully cannulated, the 8 F sheath on the contralateral iliac limb side was upgraded to a 14 F sheath (Cook, Bloomington, IN, USA). To maintain access to the aneurysm sac, a 0.035-inch hydrophilic wire (Terumo, Tokyo, Japan) was introduced into the sac via the same sheath. Even with the 0.035-inch wire parallel to the main wire inside the 14 F sheath, the 12 F contralateral iliac limb prosthesis could readily pass through.

**Fig. 1:**
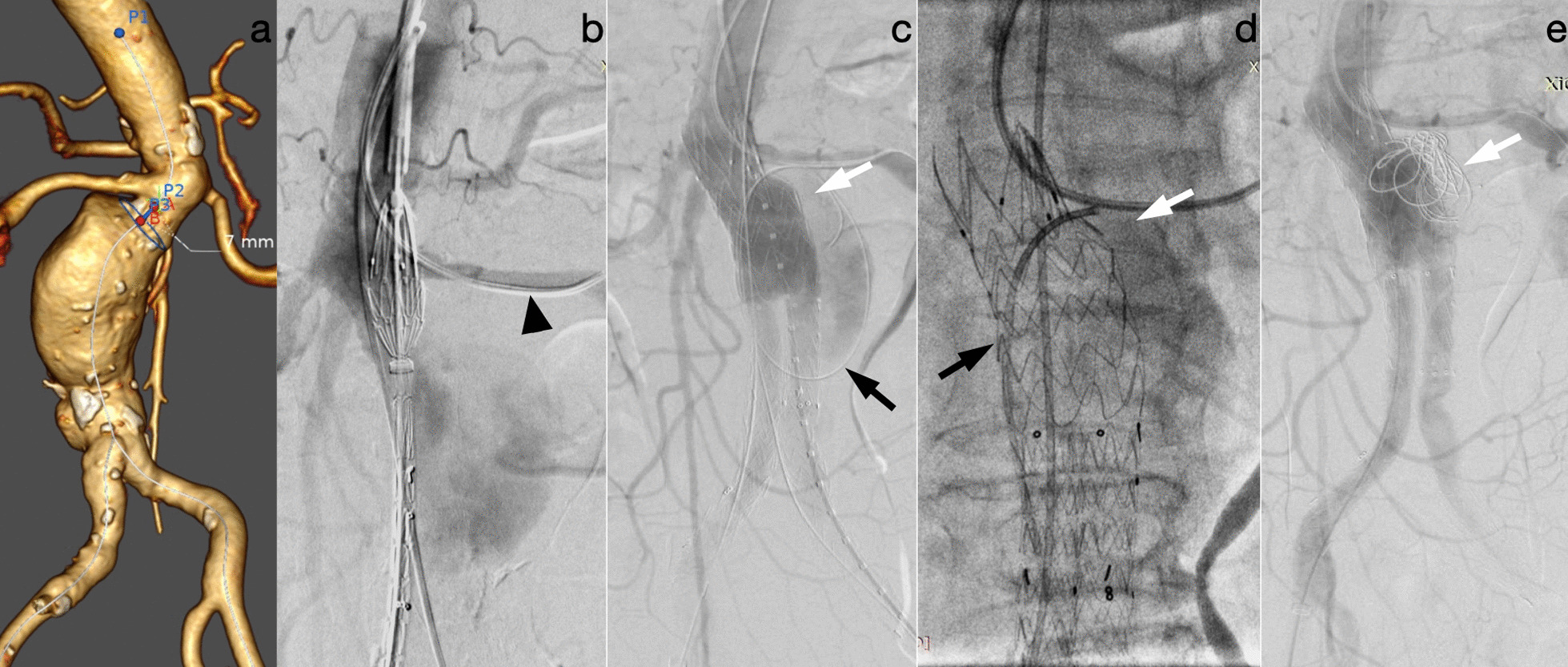
Images of a 77-year-old man who underwent EVAR for the treatment of an AAA with a hostile neck anatomy (aortic neck length, 7 mm). **a** Three-dimensional reconstruction of CTA demonstrating the short neck length of the aneurysm. **b** A 5 F catheter (arrowhead) was inserted into the left renal artery via brachial artery access to avoid unintentionally covering the left renal artery. **c** A 0.035-inch wire was preloaded into the sac (black arrow), and after endograft implantation and balloon moulding, angiography revealed a type Ia endoleak (white arrow). **d** A 5 F catheter (black arrow) was inserted via the preloaded wire into the sac neck, and saccography confirmed the catheter's position (white arrow). **e** The coils (white arrow) were delivered via a catheter to the site of the sac neck, and the final angiography confirmed resolution of the endoleak

Following complete deployment of the endograft and occlusion of the aneurysm, angiography was performed. If no ELIa persisted, the wire was retracted. If ELIa continued to persist after proximal balloon moulding, then cuff placement was performed if there was still a sufficient length of aneurysm neck. If the placement of a proximal cuff was impeded by a short landing zone or if type Ia endoleak persisted despite cuff extension, sac neck-targeted coil embolization was performed. A 5 F vertebral catheter (Terumo, Tokyo, Japan) was introduced through the preloaded wire into the sac neck region, and detachable coils (Interlock, Boston Scientific, MA, USA) were delivered through the catheter to seal the neck (Fig. 1). After successful embolization, the catheter and wire were removed. The final angiography confirmed the absence of ELIa and procedure-related type Ib or type III endoleak.

### Outcome and definitions

The primary outcomes were freedom from sac expansion and endoleak-related reinterventions during the follow-up period; secondary outcomes included technical success and intraoperative and in-hospital postoperative complications.

The status of the aortic aneurysm sac during follow-up was categorized as follows: sac regression, defined as a decrease in maximal AAA diameter ≥ 5 mm; sac expansion, defined as an increase in diameter ≥ 5 mm; and sac stability, defined as any change < 5 mm compared to the preoperative measurement. Freedom from endoleak-related reintervention was defined as the absence of endoleak during follow-up or sac growth of less than 5 mm.

Major complications included aneurysm rupture during the procedure, distal embolization, coil placement outside of the sac, procedure-related type Ib or III endoleak, puncture site haemorrhage, haematoma, and pseudoaneurysm.

**Fig. 2:**
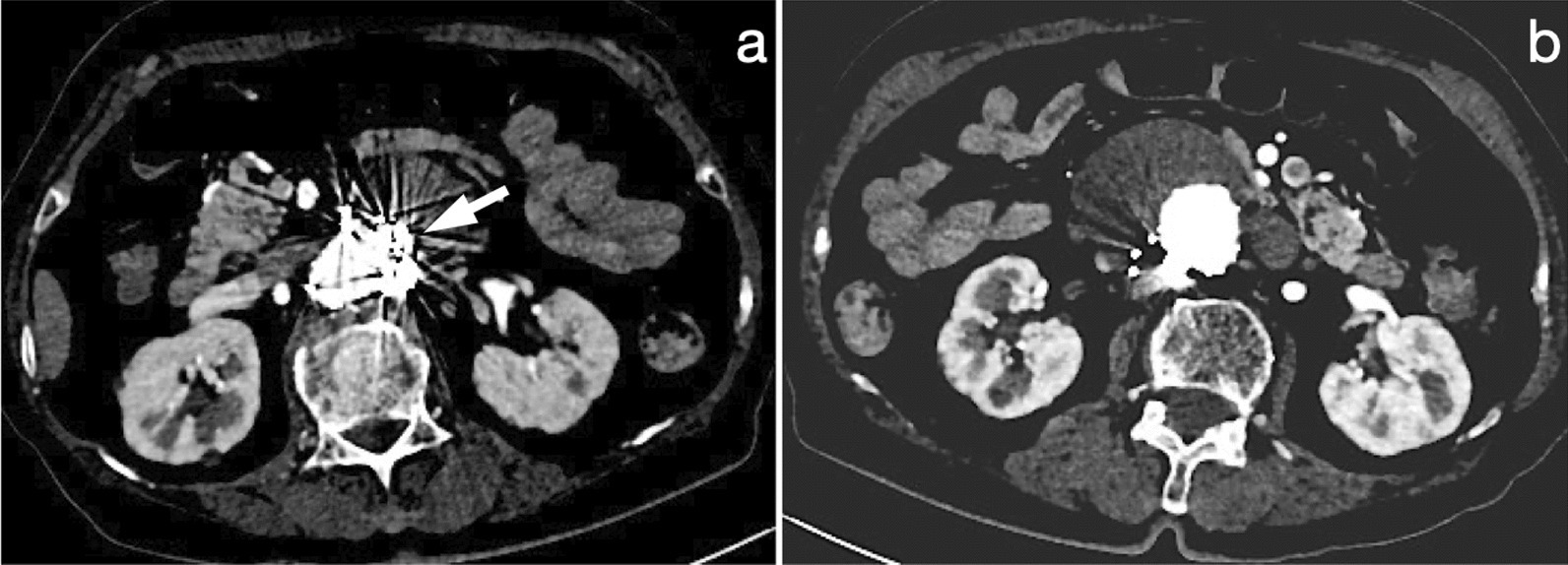
CTA during follow-up. **a** CTA 1 year after EVAR revealed no endoleak, and coils (white arrow) were placed around the sac neck. **b** There was no endoleak, and a sac regression of 7 mm was observed

All patients underwent routine surveillance contrast-enhanced CT 30 days after the initial procedure, 12 months later, and yearly thereafter for up to 5 years to monitor for endoleak and measure the diameter of the AAA sac (Fig. 2). Annually thereafter, duplex ultrasound or contrast-enhanced CT may be utilized, depending on the patient's condition.

### Data analysis

Descriptive statistics, including the frequency distribution, mean, and standard deviation, were used to analyse the baseline data and follow-up results. The Shapiro–Wilk test was applied to evaluate the normality of data distributions. Continuous data are reported as the mean with standard deviation (SD) or median with range. If data were not normally distributed, nonparametric analysis methods were applied to analyse them. Categorical data are presented as numbers (n) and percentages (%), and chi-squared or Fisher's exact test was used to compare categorical variables. MedCalc software (MedCalc Software Ltd., Ostend, Belgium) was used for statistical analyses, and *P* values less than 0.05 were considered statistically significant.

## Results

A total of twenty-eight patients underwent elective occlusion of an AAA with a hostile neck anatomy using this technique for selective embolization of the aneurysmal sac neck. The coiling group consisted of 11 patients (39.29%), whereas the noncoiling group included 17 patients (60.71%). The baseline patient demographics are presented in Table 1, which revealed that male sex (78.57%), hypertension (60.71%), and smoking (53.57%) were the main factors affecting patients. No patient presented with severe renal dysfunction. There were no differences between the coiling and noncoiling groups in terms of patient characteristics.

**Table 1:** Patient characteristics

Variable	Total (N = 28)	Coiling (N = 11)	Non-coiling (N = 17)	*P* value
Age (y)	71.46 ± 8.98	70.64 ± 10.77	71.46 ± 8.98	0.83
Male (sex)	22 (78.57%)	8 (72.7%)	14 (82.35%)	0.44
BMI (kg/m^2^)	24.72 ± 3.57	24.30 ± 4.06	25.03 ± 3.34	0.61
Systolic blood pressure (mmHg)	126.36 ± 19.10	121.73 ± 14.49	128.06 ± 16.84	0.30
Diastolic blood pressure (mmHg)	75.11 ± 11.28	73.64 ± 9.96	79.06 ± 10.25	0.18
Smoking history	15 (53.57%)	5 (45.45%)	10 (58.82%)	0.38
Diabetes mellitus	6 (21.43%)	4 (36.36%)	2 (11.76%)	0.14
Hypertension	17 (60.71%)	5 (45.45%)	12 (70.59%)	0.18
Hyperlipidemia	7 (25.00%)	3 (27.27%)	6 (35.29%)	0.49
Cardiac status	5 (17.86%)	4 (36.36%)	1 (5.88%)	0.06
Renal status	7 (25.00%)	3 (27.27%)	4 (23.53%)	0.58
Pulmonary status	7 (25.00%)	2 (18.18%)	5 (29.41%)	0.42
Peripheral arterial disease	8 (28.57%)	4 (36.36%)	4 (23.53)	0.38
Lab result				
Hemoglobin (mmol/L)	129.18 ± 21.80	130.41 ± 21.77	132.41 ± 22.50	0.46
Lymphocyte (× 10^9^/L)	1.30 ± 0.59	1.19 ± 0.48	1.37 ± 0.65	0.44
Serum creatinine (μmol/L)	90.20 ± 37.08	91.69 ± 52.06	89.23 ± 24.96	0.87
GFR (ml/min/1.73 m^2^)	71.60 ± 22.46	75.97 ± 29.27	68.78 ± 17.14	0.32

Continuous data are presented as mean ± standard deviation. Categorical data are given as counts (percentages)

*GFR* glomerular filtration rate

Table 2 lists the baseline aneurysm anatomy characteristics. The mean aneurysm diameter was 59.27 ± 16.41 mm, and no patient had an unsuitable access artery morphology due to an insufficient access vessel diameter. In the coiling group, 3 patients (27.27%) had a neck angle > 60°, 1 patient (9.09%) had a neck length < 15 mm, 3 patients (27.27%) had two hostile neck characteristics, and 4 patients (36.36%) had three hostile neck characteristics. In the noncoiling group, one patient (5.88%) had a neck angle > 60°, one patient (5.88%) had a neck length < 15 mm, 12 patients (70.59%) had two hostile neck characteristics, and 3 patients (17.65%) had three hostile neck characteristics. There were no differences in the anatomical characteristics of aneurysms between the coiling and noncoiling groups.

**Table 2:** Baseline characteristics of aneurysmal anatomy

Data	Total (N = 28)	Coiling (N = 11)	Non-coiling (N = 17)	*P* value
Anatomic data (mean ± SD), mm				
Proximal aortic neck diameter	22.17 ± 3.29	24.20 ± 4.68	21.18 ± 1.81	0.09
Proximal aortic neck length	22.37 ± 7.26	21.61 ± 4.88	22.8 ± 9.44	0.68
Proximal aortic neck angulation	50.63° ± 27.15°	53.44° ± 31.25°	50.79 ± 26.86	0.94
Smaller EIA diameter	9.36 ± 2.21	10.24 ± 1.34	8.94 ± 2.57	0.20
AAA diameter	59.27 ± 16.41	60.98 ± 18.61	58.81 ± 16.56	0.89
Distribution of HNA				
Neck angle > 60°	4 (14.29%)	3 (27.27%)	1 (5.88%)	0.14
Neck length < 15 mm	2 (7.14%)	1 (9.09%)	1 (5.88%)	0.14
Conical neck	0	0	0	–
Calcification ≥ 50%	0	0	0	–
Thrombus ≥ 50%	0	0	0	–
Multiple HNA characteristics:	22 (78.57%)	7 (63.63%)	15 (88.24%)	
2 characteristics	15 (53.57%)	3 (27.27%)	12 (70.59%)	0.14
3 characteristics	7 (25.00%)	4 (36.36%)	3 (17.65%)	0.14

Continuous data are presented as mean ± standard deviation. Categorical data are given as counts (percentages)

*EIA* external iliac artery, *AAA* abdominal aortic aneurysm, *HNA* hostile neck anatomy

All procedures were performed percutaneously, and local anaesthesia was administered to 17 patients (60.71%), including 6 patients (54.54%) in the coiling group and 11 patients (64.70%) in the noncoiling group. Table 3 lists the details of the intraoperative parameters and outcomes. Technical success was achieved in every case, and no intraoperative complications, such as aneurysm rupture, distal embolization, coil placement outside the sac, or type Ib or type III endoleak, were observed. The coiling group showed longer operation times (81.27 ± 11.61 vs. 70.71 ± 7.17 min, *P* < 0.01) and greater contrast utilization than the noncoiling group (177.45 ± 52.41 vs. 108.24 ± 17.49 ml, *P* < 0.01), with most of the coil procedure time spent waiting a few minutes after each coil embolization to evaluate the extent of the endoleak. All patients in the coiling group underwent balloon moulding, compared to 14 patients in the noncoiling group (100% vs. 82.35%, *P* = 0.26). In the coiling group, there were two instances of cuff extension (with the diameter of the cuff the same as that of the endograft), compared to none in the noncoiling group (18.18% vs. 0, *P* = 0.15). The coil ranged in size from 12 to 20 × 40 cm, and the mean number of coils used was three (range 2–4).

**Table 3:** Intraoperative parameters and outcomes

Data	Total (N = 28)	Coiling (N = 11)	Non-coiling (N = 17)	*P* value
Anesthesia				
Region	17 (60.71%)	6 (54.54%)	11 (64.70%)	0.70
Vascular access				
Common Femoral artery puncture	28(100%)	11(100%)	17(100%)	–
Time of operation (min)	74.86 ± 10.39	81.27 ± 11.61	70.71 ± 7.17	0.006*
Amount of contrast (mL)	135.43 ± 48.82	177.45 ± 52.41	108.24 ± 17.49	< 0.0001*
Blood loss (mL)	121.35 ± 20.05	125.79 ± 21.63	118.92 ± 19.81	0.39
Adjunctive treatment				
Balloon molding	25 (89.29%)	11 (100%)	14 (82.35%)	0.26
Cuff extension	2 (7.14%)	2 (18.18%)	0	0.15
Technical success	100%	100%	100%	–
major complications				
Aneurysm rupture	0	0	0	–
Distal embolization	0	0	0	–
Endoleaks^a^	0	0	0	–
Immediate and 30-day results, n (%)				
Length of stay (days)	7.27 ± 1.01	7.45 ± 1.21	7.17 ± 0.99	0.49
Puncture site complications^b^	0 (0.00%)	0 (0.00%)	0 (0.00%)	–
Acute limb ischemia	0 (0.00%)	0 (0.00%)	0 (0.00%)	–
1-year follow-up				
Sac regression	19 (67.86%)	7 (63.64%)	12 (70.59%)	1.00
Sac stability	9 (32.14%)	4 (36.36%)	5 (29.41%)	1.00
Sac expansion	0 (0.00%)	0 (0.00%)	0 (0.00%)	–
Endoleak-related reintervention	0 (0.00%)	0 (0.00%)	0 (0.00%)	–

Continuous data are presented as mean ± standard deviation. Categorical data are given as counts (percentages)

*Significant difference between subgroups (*P* < 0.05)

^a^Type Ia, Ib and III

^b^Puncture site hemorrhage, hematoma, and pseudoaneurysm

All included patients had in-hospital and follow-up data for analysis. At the 30-day time point, there were no cases of complications at the puncture site or acute limb ischaemia. The median duration of follow-up was 18.6 ± 5.2 months, ranging from 12 to 31 months. All patients were completely free of sac expansion (19 cases of sac regression, 67.86%; 9 cases of sac stability, 32.14%) and endoleak-related reintervention. In the coiling group, there were 7 patients (63.64%) with sac regression and 4 patients (36.36%) with sac stability, while in the noncoiling group, there were 12 patients (70.59%) with sac regression and 5 patients (29.41%) with sac stability; no statistically significant differences were found between the groups.

## Discussion

Numerous studies have demonstrated that standard EVAR for AAA patients with an HNA can nevertheless achieve satisfactory long-term outcomes, confirming the efficiency of EVAR in cases of unfavourable morphologies [15, 16]. However, the increased incidence of ELIa has been linked to a hostile neck anatomy due to an insufficient seal between the aortic wall and the endograft attachment sites [8, 17]. Although fenestrated stent grafting can serve as an effective endovascular solution for some of these patients [18], it is currently not available off-the-shelf in China.

This retrospective study assessed the short-term efficacy and safety of intraprocedural selective coil embolization of the aneurysmal sac neck to eliminate persistent ELIa in the endovascular repair of AAAs with a hostile neck morphology. The findings of the study are promising. In cases where there is a persistent ELIa, coil embolization can be effectively carried out in the perigraft sac neck region to address this issue. On the other hand, in cases where there is no ELIa, the preloaded wire can be retracted without any complications related to the procedure. The results of the 1-year follow-up indicate that there was a 100% absence of sac expansion and endoleak-associated reintervention, despite the challenging clinical and anatomic conditions of the patients.

The necessity of treating ELIa at completion angiogram in endovascular repair, also referred to as primary ELIa, is the subject of some controversy. Several publications in the literature have indicated that a persistent primary ELIa may resolve spontaneously in the majority of cases during the follow-up period [19, 20]. In contrast, several publications have shown that primary ELIa is associated with increased all-cause and aneurysm-related mortality [21, 22], and current guidelines recommend intraoperative adjunctive therapies in the case of ELIa identification [2, 23].

Recent reports have described the increasing use of prophylactic intraoperative embolization of the aneurysm sac with glue, coils, thrombin, and N-butyl cyanoacrylate (NBCA) or a combination of these to prevent type II endoleak and improve sac regression [11, 12, 24]. Studies on ELIa treatment through embolization have typically focused on postprocedural treatment [25, 26], targeting the area between the endograft and aneurysmal neck. Ameli-Renani et al. [27] reported the utilization of transcatheter embolization to treat 23 patients diagnosed with ELIa post-EVAR. This technique demonstrated 80% freedom from endoleak recurrence and 85% freedom from sac growth over an average of 311 days (1–1357 days) of follow-up. In addition, 5 years later, Patel et al. [28] from the same team published a study on 27 patients with ELIa using the same technique and longer follow-up results (mean, 25 months). They concluded that transcatheter embolization for ELIa is a safe and effective option in a select patient cohort where traditional endovascular options are unsuitable or have failed.

However, reports of intraprocedural embolization for ELIa when adjunctive treatment is unavailable or ineffective are rare [29, 30]. Marchiori et al. [31] reported that embolization for ELIa was successful in 22 patients following EVAR and that four patients were treated during the standard index EVAR procedure. Acceptable clinical and radiologic outcomes were observed during the 24-month follow-up period; however, no distinction was made between patients who underwent embolization during the index procedure or reinterventions. Femoral or upper extremity access was used to reach the embolization site, and the catheter or, on occasion, an additional microcatheter was needed to navigate within the sac neck [29–31]. During catheter engagement of the endoleak location, this procedure was potentially time-consuming and carried a risk of causing an aortic dissection.

While performing the EVAR procedure, it is difficult to predict the incidence of ELIa due to its dependence on the patient's aortic anatomy and stent graft utilization. A hostile neck anatomy encompasses a spectrum of risk factors, and there is a scarcity of evidence specifying which configuration is the most prevalent cause of intraoperative ELIa [32, 33]. Our study, with a small sample size, demonstrated that there was no difference in baseline patient characteristics or aortic neck morphology between the coiling and noncoiling groups, highlighting the benefit of the selective aneurysmal sac neck-targeted embolization technique in the case of intraoperative ELIa. Compared to the catheter technique for accessing the endoleak cavity from within the endograft, this alternative technique is a safer and more convenient option for addressing persistent ELIa.

Embolic agents consist of coils, plugs, liquid embolic agents (e.g., NBCA), or a combination of these components. Compared with coils and plugs, the use of a liquid embolic agent requires a relevant learning curve [31, 34]. Because we were focusing on the region between the sac neck and endograft, it was prudent to utilize NBCA in the event that the liquid embolic agent dislodged beyond the aneurysm sac. While the majority of publications have demonstrated the safety of using NBCA for ELIa treatment, there has been one report of a complication involving the reflux of onyx into one of the graft limbs, resulting in notable stenosis. Due to our limited familiarity with NBCA, we utilized coils exclusively. According to a study by Mascoli et al. [35], the effectiveness of the embolization procedure in preventing type II endoleak can be enhanced by augmenting the concentration of the implanted coils. Specifically, the neck-targeted embolization technique can produce a significant concentration of coils in the region surrounding the perigraft sac neck.

However, coils are associated with artefacts on CT angiography, which might bias any assessment of the endoleak. Our surveillance protocol for patients undergoing coil embolization included CT angiography, with a primary focus on the maximal AAA diameter. If endoleak or sac expansion is suspected, further diagnostic procedures, such as digital subtracted angiography or duplex ultrasound, will be applied to confirm the diagnosis.

Our study also demonstrated that if there was no endoleak after endograft deployment or adjunctive treatment, the preloaded wire did not induce the rupture of the aneurysm, dislodgement of the thrombus within the sac, or procedure-related type Ib or type III endoleak, as it minimally impeded the implantation of the iliac limb prosthesis. The upgraded sheath, which enabled the concurrent passage of the iliac limb prosthesis and parallel wire, did not result in any puncture site complications during percutaneous EVAR.

This research has several limitations. First, it was a retrospective study with a small sample size and the need for extended follow-up to assess the technique's efficacy in terms of late postoperative aneurysm shrinkage and freedom from endoleak-related reintervention. In addition, a prospective randomized study may be needed to validate this method in future research.

## Conclusions

The prophylactic preloading of a wire into the sac as a backup to perform selective aneurysmal sac neck-targeted embolization is feasible and safe and can increase the probability of successfully resolving potential instances of ELIa intraoperatively in cases where traditional endovascular options are unsuitable or have failed.

## Data Availability

All patient information is stored in the electronic record system and the PACS system of the four institutions; if necessary, we can provide details of each patient without revealing their private information.
